# A first case report of nasopharyngeal *Mycobacterium abscessus subspecies massiliense* infection

**DOI:** 10.1186/s40001-021-00578-8

**Published:** 2021-09-18

**Authors:** Yamato Oki, Hiromitsu Hatakeyama, Masanori Komatsu, Yasuhiro Isono, Hidetaka Ikemiyagi, Jun Tsukiji, Ryoko Higa, Nobuhiko Oridate

**Affiliations:** 1 Department of Otolaryngology, Yokohama City University Medical Center, Urafune-cho 4-57, Minami-ku, Yokohama, 232-0024 Japan; 2 Department of Infectious Disease, Yokohama City University Medical Center, Urafune-cho 4-57, Minami-ku, Yokohama, 232-0024 Japan; 3 Department of Otolaryngology, Yokohama City University Graduate School of Medicine, 3-9 Fukuura, Kanazawa-ku, Yokohama, 236-0004 Japan

**Keywords:** *Mycobacterium massilliense*, *Mycobacterium abscessus subspecies massiliense*, Nasopharyngitis, Non-tuberculosis mycobacterium, NTM, *Mycobacterium abscessus* complex, Case report

## Abstract

**Background:**

*Mycobacterium abscessus subspecies massiliense* is a non-tuberculous mycobacteriosis and was subdivided from *Mycobacterium abscessus* in 2006. This article is the first report on nasopharyngitis caused by *Mycobacterium abscessus subspecies massiliense*.

**Case presentation:**

A 45-year-old woman had an 18-month history of recurrent nasopharyngitis and presented with pain in the throat. Mycobacterial tissue culture and polymerase chain reaction testing revealed the presence of *Mycobacterium abscessus subspecies massiliense* in the nasopharyngeal tissue. This patient underwent surgery, followed by multiple rounds of chemotherapy with oral and intravenous antibiotic agents for 16 weeks. She has had no recurrence during the 56 weeks since treatment.

**Conclusion:**

It is difficult to detect the presence of *Mycobacterium abscessus subspecies massiliense* in a culture from the swabbing sample. The tissue culture from a biopsy specimen is mandatory for the identification of the species. Currently, no definite treatment policy is available and only empirical treatment is applied. This case is an important for the diagnosis and treatment of this bacterial infection on nasopharynx.

## Background

*Mycobacterium abscessus subspecies massiliense* (referred to hereafter as *M. massiliense*) is a non-tuberculous mycobacterosis (NTM) and is included among the rapidly growing mycobacteria (RGM) [1]. It was subdivided from *Mycobacterium abscessus (M. abscessus*) in 2006 [2].

*M. massiliense* is rare species described in only a few reports [3, 4]. The *M. abscessus* complex, including *M. massiliense*, commonly causes skin, soft tissue as well as pulmonary infections [5]. Although there are reports of *M. massiliense* infection in the head and neck region, such as otitis media [6], there are no reports of nasopharyngitis caused by this pathogen. We here report, to our knowledge, the first case of nasopharyngeal *M. massiliense* infection.

## Case presentation

A 45-year-old woman was referred to Yokohama City University Medical Center with an 18-month history of recurrent nasopharyngitis. She presented with pain in the throat. Nasopharyngitis had been identified on a previous visit to an otolaryngology clinic. It had been temporarily improved by antibacterial treatment and nasopharyngeal abrasive therapy, which involves rubbing the nasopharynx with a nasal swab or pharyngeal cotton thread soaked in zinc chloride solution or compound iodine glycerin [7, 8]. However, it recurred within a few weeks, and nasopharyngeal abrasive therapy continued for 12 weeks before the first visit. She was then referred to our hospital for further examination and treatment.

**Fig. 1:**
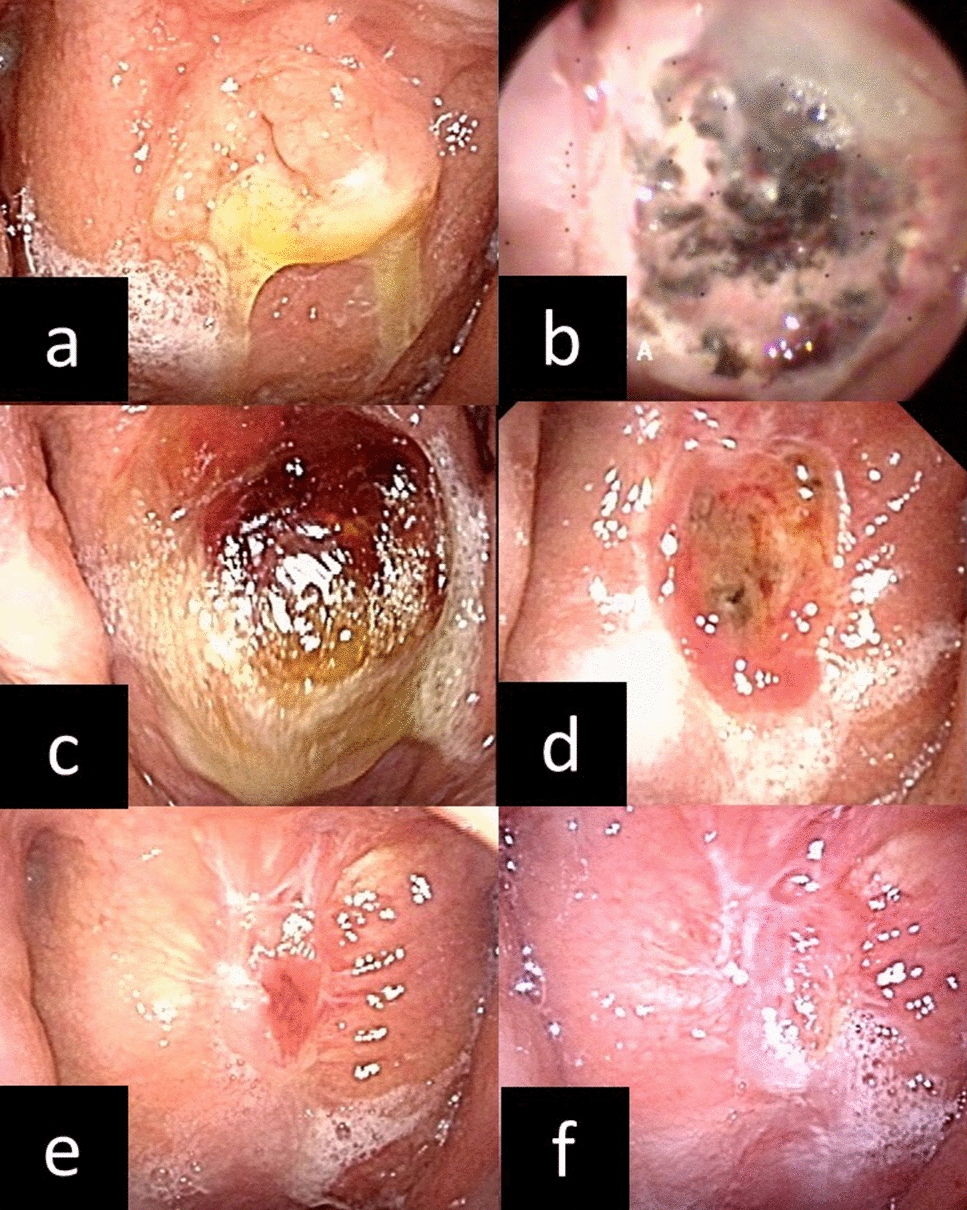
**a** Findings of the nasopharynx before treatment. There were a raised nasopharyngeal lesion and a pus on the lesion. **b** Findings of the nasopharynx after adenoidectomy. Fur and black scabs for hemostasis had been attached to the wound. **c** 2 weeks later after surgery. There was a pus on the lesion. The pus on wound was appropriately removed by dealing and washed. **d** 4 weeks later after surgery. There was granulation rising in the wound. **e** 12 weeks later after surgery. The granulation remained but the wound was smaller gradually. **f** 20 weeks later after surgery. The wound is epithelialized and scarred

At presentation, she had infectious swelling of the adenoid remnant and pus on the lesion (Fig. 1a). Her physical examination was otherwise unremarkable and she had no other medical history including immunodeficiency.

**Fig. 2:**
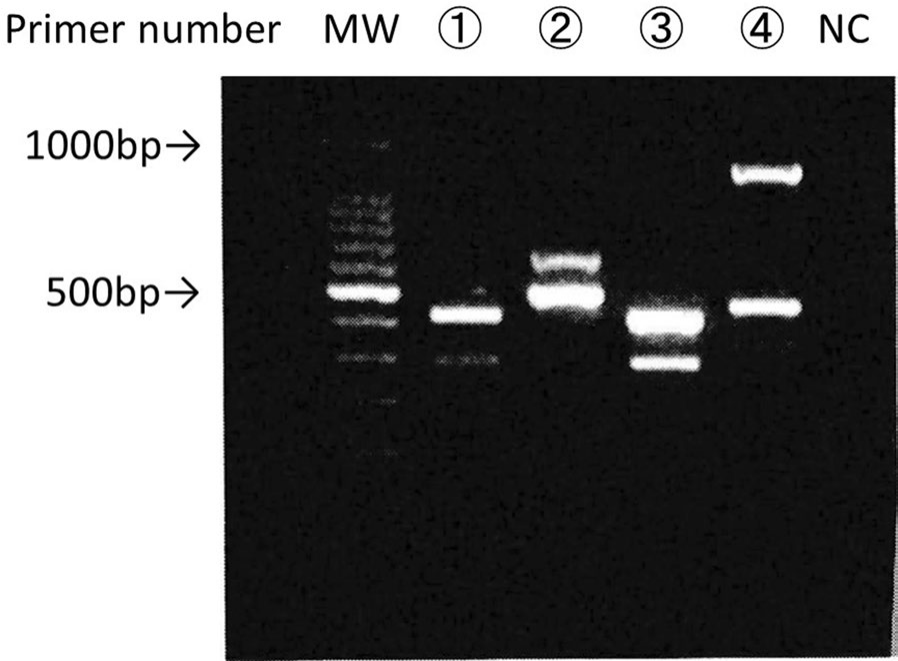
Examination showed the disease as *Mycobacterium abscessus subspecies massiliense* infection. MW: molecular weight, ① Sample of this case, ② *Mycobacterium abscessus subspecies abscessus*, ③ *Mycobacterium abscessus subspecies massiliense*, ④ *Mycobacterium abscessus subspecies bolletii*. *NC* negative control

The first pharyngeal swab culture test, which was gotten from epipharynx to insert the swab through the anterior nasal cavity, showed normal flora at the time of first visit. The same day, histopathological examination of a nasopharyngeal biopsy sample revealed the possibility of mycobacterial infection based on positive results for acid-fast bacillus and Ziehl–Neelsen staining. Three weeks later, the first examination by pharyngeal mycobacteria culture from the sample which gotten by swabbing was positive for smear dyeing of acid-fast bacillus stain, while the culture was negative. *Four weeks later from first visit, we got the second mycobacterial culture from the biopsy specimen collected from nasopharynx using the sterile nasal cupped forceps with the visual guide of endoscope. It was wrapped in sterile gauze moistened with raw food to avoid exposure to tap water and transported promptly to the laboratory.* It revealed the presence of *M. abscessus* complex using the MGIT™ System. (Becton and Dickinson Co. Mountain View, CA) All strains were classified as *M.* *abscessus* complex using a DDH Mycobacteria kit (Kyokuto Pharmaceutical Industrial, Tokyo, Japan) or by MALDI-TOF MS (Bruker Daltonics, Billerica, MA, USA). *Mycobacterium abscessus subspecies abscessus*, *M. massiliense* and *Mycobacterium abscessus subspecies bolletii* type strains were obtained from the Japan Collection of Microorganisms of the Riken Bio-Resource Center (BRC-JCM; Ibaraki, Japan). All bacterial strains/isolates were subcultured on 2% Ogawa egg slants or 7H10 agar plates supplemented with 10% OADC*. All procedures were performed in a laminar flow cabinet to avoid contamination.* PCR assays were done for differentiating *Mycobacterium abscessus subspecies abscessus*, *M. massiliense*, and *Mycobacterium abscessus subspecies bolletii,* as described previously [9], 10, and identified this species as *M. massiliense* (Fig. 2). The susceptibility test was not performed this case. This is because there is a difference between the in vitro results and the behaviors under antimicrobial use, and the information is for limited reference only [1, 10].

**Fig. 3:**
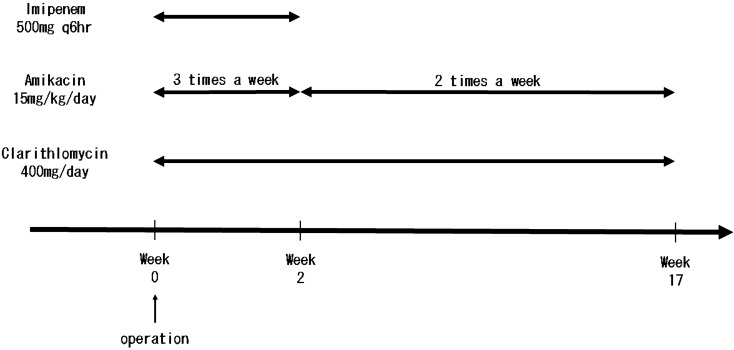
Patient was performed the operation, and at the same time, was treated with clarithromycin, imipenem and amikacin at intervals of three times a week. She was received with the multiple antibacterial drugs for 2 weeks. After 2 weeks of the hospitalization, she was treated with clarithromycin and amikacin at intervals of two times a week for 16 weeks

The patient underwent surgery to remove the inflamed adenoid tissue in the nasopharynx and washed the wound with saline. At the same time, she was treated with oral clarithromycin at 400 mg/day, intravenous imipenem at 2 g/day divided into four doses, and intravenous amikacin at 15 mg/kg/day three times a week. She received multiple antibacterial therapy for 2 weeks in an inpatient setting. She was then treated with oral clarithromycin at the same dosage, and intravenous amikacin two times a week after discharge. The treatment courses are summarized in Fig. 3. She received this combination therapy with clarithromycin and amikacin for an additional 4 months. The mycobacterial “tissue” culture was negative 8 weeks after surgery. She is currently being followed up in our hospital, and has had no adverse event and no recurrence during the 56 weeks since the cessation of treatment.

## Conclusions and discussion

### Disease from *M. massiliense* and its infectious route

Due to the low incidence of mycobacterial infections themselves, statistically, while there is a lot of information on the pulmonary region of *M. massiliense*, which accounts for about 0.6% of all NTM infections, *M. massiliense* infections in the head and neck region have rarely been reported [4]. As far as we are aware, this patient is the first case of pharyngeal *M. massiliense* infection.

It has been reported that infections caused by the *M. abscessus* complex usually follow accidental trauma or surgery [11]. On the other hand, multiple iatrogenic cases of NTM infection have been described in the past 2 decades [1], including cases resulting from cardiac surgery, injections, plastic surgery, middle ear tympanostomy tube replacement, and a variety of miscellaneous surgical procedures [1, 12–18]. Most of the health care-associated mycobacterial outbreaks have involved RGM, especially the *M. abscessus* complex and *Mycobacterium fortuitum* [19]. The common factor in health care-associated outbreaks is presumed to be exposure of a susceptible individual to an NTM-infected liquid, usually tap water [1]. As it may have been overlooked due to a low detection rate in culture tests, although NTM infection is rare its possible presence must still be considered.

The *M. abscessus* complex is also frequently detected in water and soil, and, in general, there are many cases of skin and soft tissue infections associated with trauma or surgery [11, 20].

NTMs are widely distributed in the environment [21, 22], and it is considered that NTM infection in humans is caused by bacterium from the environment [4]. As NTMs originally have only weak pathogenicity in humans, it parasitizes and infects humans. Therefore, infections are caused by some trigger, such as immunodeficiency [4]. On the other hand, some patients without obvious immunodeficiency develop NTM disease [23].

This case was a 45-year-old, previously healthy woman who was not immunodeficient. She should, therefore, be less likely to get infected. However, she had undergone nasopharyngeal abrasive therapy for 12 weeks prior to visit to our hospital. The purpose of this therapy is sterilization and disinfection using a coating agent and phlebotomy [7, 8]. It is said that the greater the degree of disinfection, the higher the amount of bleeding, but the more effective it is. It is possible that the patient, who was not immunodeficient, was infected by *M. massiliense,* because she had been repeatedly scratched around the nasopharynx with a machine using tap water.

### Diagnosis

As NTM infections are very rare in the head and neck area, NTM infections are not often expected as a cause of nasopharyngitis. There may be more cases of pharyngeal NTM infections involved with pharyngitis that is resistant to treatment. We recommend that tissue samples are collected actively for the assessment of pharyngeal NTM infections where possible. It is important to preserve the quality and quantity of the samples for diagnosis [24]. *Specifically, it is necessary to collect a sufficiently large specimen volume and to avoid exposure to bacteria from the surrounding environment, especially tap water, during collection. It is also a good idea to protect the specimen submission with sterile gauze soaked in reproduction to prevent it from drying out.*

In this case, *M. massiliense* could not be identified by swab at first, and the biopsy sample could identify *M. abscessus* complex by culture examination. Although it may be difficult to obtain specimens from other parts of the body, such as the lungs, it is not difficult to obtain specimens from the head and neck region. Therefore, early biopsy is recommended for the diagnosis.

Because of differences in antimicrobial susceptibility in NTM infections, determining treatment policy is problematic, and species-level identification of the NTM is becoming increasingly important on a clinical level [24]. The PRA (Polymerase Chain Reaction-restriction enzyme pattern analysis) method identifies many NTM species that are not identifiable by phenotypic or chemotaxonomic techniques alone [1].

### Treatment

*M. abscessus* complex isolates are uniformly resistant to standard anti-tuberculous agents [25–27] According to the American Thoracic Society/Infectious Diseases Society of America (ATS/IDSA), there is no reliable antibiotic regimen to produce a cure for the *M. abscessus* complex at present [1]. However, periodic administration of multidrug therapy, including a macrolide and one or more parenteral agents or a combination of parenteral agents over several months, may help control symptoms and the progression of *M. abscessus* complex disease [1]. In addition, it is reported that the only predictably curative therapy for focal *M. abscessus* complex disease is surgical resection combined with multidrug chemotherapy [1]. The treatment is limited to the current treatment policy for lung infections due to the problem with a lack of accumulated evidence.

The ATS/IDSA recommends the following chemotherapy for serious skin, soft tissue, and bone infections caused by the *M. abscessus* complex. Clarithromycin or azithromycin should be combined with parenteral medications (amikacin, cefoxitin, or imipenem) [26, 28]. The macrolides are the only oral agents found to be reliably active in vitro against the *M. abscessus* complex [26, 28].

The most active of the parenteral agents is amikacin [29]. Amikacin combined with high-dose cefoxitin is recommended for the initial therapy (minimum, 2 weeks) [25, 26, 30]. Therefore, hospitalization is recommended for a minimum of 2 weeks. However, limited cefoxitin availability may necessitate the choice of an alternative agent, such as imipenem [25, 26, 30]. For serious disease, a minimum of 16 weeks of therapy is necessary to increase the likelihood of a cure [1].

The clinical identification of *M. massilience* is significant due to the difference in therapeutic response between *M. massiliense* and other species [20]. A relatively large number of reports have described that even if treatment with the same treatment regimen is performed, the improvement rate in clinical findings, including imaging and physical findings, and symptoms are be better in *M. massiliense* than in other species [31–33]. The reason for the difference in response is the erm gene, which is a resistance-inducing gene for macrolide [34]. Thus, it may take less time to cure *M. massiliense* infection than other species [32].

In this case, treatment was performed according to the ATS/IDSA. Therefore, we did not use tigecycline. As the patient was exhausted by a few months of outpatient treatment, we had a discussion about the treatment policy with a doctor specializing in infectious diseases, including whether or not to switch to oral medicine. However, we decided to continue conventional treatment as far as possible. Eventually, the patient could not tolerate the outpatient treatment, and treatment ended after 16 weeks. It is possible to provide treatment that emphasizes the patient’s quality of life if a shorter treatment period or an oral treatment regimen that is easy to continue is available that affords a better improvement rate in *M. massiliense* infections.

We reported the first case of pharyngeal *M. massiliense* infection. There may be more cases of pharyngeal NTM infections among cases of pharyngitis that are resistant to therapy. This patient underwent surgery and the multiple rounds of chemotherapy with oral and intravenous agents for 16 weeks, and no recurrence has been observed for the past 56 weeks. There is currently no definite treatment policy, and only empirical treatment is used; therefore, the further accumulation of data is expected in the future.

## Data Availability

Not applicable.

## Abbreviations

ATS/IDSA: American Thoracic Society/Infectious Diseases Society of America
*M. abscessus*: *Mycobacterium abscessus*
*M. massiliense*: *Mycobacterium abscessus subspecies massiliense*
NTM: Non-tubeculous mycobacteria
PCR: Polymerase chain reaction
RGM: Rapidly growing mycobacteria
